# Objective discrimination of bimodal speech using frequency following responses

**DOI:** 10.1016/j.heares.2023.108853

**Published:** 2023-07-09

**Authors:** Can Xu, Fan-Yin Cheng, Sarah Medina, Erica Eng, René Gifford, Spencer Smith

**Affiliations:** a Department of Speech, Language, and Hearing Sciences, University of Texas at Austin, 2504A Whitis Ave. (A1100), Austin 78712-0114, TX, USA; b Department of Speech, Language, and Hearing Sciences, Vanderbilt University Medical Center, Nashville, TN, USA

**Keywords:** Cochlear implants, Hearing aids, Bimodal benefits, Frequency following responses, Machine learning

## Abstract

Bimodal hearing, in which a contralateral hearing aid is combined with a cochlear implant (CI), provides greater speech recognition benefits than using a CI alone. Factors predicting individual bimodal patient success are not fully understood. Previous studies have shown that bimodal benefits may be driven by a patient’s ability to extract fundamental frequency (f0) and/or temporal fine structure cues (e.g., F1). Both of these features may be represented in frequency following responses (FFR) to bimodal speech. Thus, the goals of this study were to: 1) parametrically examine neural encoding of f0 and F1 in simulated bimodal speech conditions; 2) examine objective discrimination of FFRs to bimodal speech conditions using machine learning; 3) explore whether FFRs are predictive of perceptual bimodal benefit. Three vowels (/*ε*/, /i/, and /ʊ/) with identical f0 were manipulated by a vocoder (right ear) and low-pass filters (left ear) to create five bimodal simulations for evoking FFRs: Vocoder-only, Vocoder +125 Hz, Vocoder +250 Hz, Vocoder +500 Hz, and Vocoder +750 Hz. Perceptual performance on the BKB-SIN test was also measured using the same five configurations. Results suggested that neural representation of f0 and F1 FFR components were enhanced with increasing acoustic bandwidth in the simulated “non-implanted” ear. As spectral differences between vowels emerged in the FFRs with increased acoustic bandwidth, FFRs were more accurately classified and discriminated using a machine learning algorithm. Enhancement of f0 and F1 neural encoding with increasing bandwidth were collectively predictive of perceptual bimodal benefit on a speech-in-noise task. Given these results, FFR may be a useful tool to objectively assess individual variability in bimodal hearing.

## 1. Introduction

Recent research has shown that 72-77% of new adult cochlear implant recipients have aidable low frequency acoustic hearing in the non-implanted ear that can be used in a bimodal hearing configuration (Holder et al., 2018; Patro et al., 2022). Bimodal hearing benefits for the average patient have been demonstrated in a variety of auditory domains including: speech perception in quiet and noise (Dorman et al., 2008; Kiefer et al., 2005; Mok et al., 2006; Sheffield et al., 2015; Sheffield and Gifford, 2014; Zhang et al., 2010), localization and spatial hearing (Ching et al., 2004; Dunn et al., 2005; Gifford et al., 2014; Potts et al., 2009), as well as music perception and appreciation (Crew et al., 2016; D’Onofrio et al., 2019, 2020; D’Onofrio and Gifford, 2021; Dorman et al., 2008; El Fata et al., 2009; Kong and Carlyon, 2007; Tawdrous et al., 2022). However, factors predicting an individual bimodal patient’s success are less clear (Ching et al., 2007; Gifford and Dorman, 2019). For example, audiometric configuration only weakly accounts for bimodal hearing performance, and this relationship appears to be driven by patients with mild (i.e., large bimodal benefit) and profound (i.e., minimal or no bimodal benefit) hearing loss but not by those with audiometric thresholds in the moderate-to-severe range (Blamey et al., 2015; Illg et al., 2014; Kessler et al., 2020b; Mok et al., 2006; Seeber et al., 2004; Zhang et al., 2013). Similarly, performance on a variety of psychoacoustic assessments targeting temporal and/or spectral resolution have mostly failed to predict bimodal benefit at the individual level or may be broadly unsuitable for clinical use given their protracted test times (e.g., Kessler et al., 2020b; Zhang et al., 2013). Together, these observations highlight a current need to develop more sophisticated tools that better account for individual variability in bimodal benefit and inform clinical decision making on a single-patient basis.

Recent perceptual studies of bimodal patients and simulations with normal hearing listeners have demonstrated that bimodal benefit can occur with the addition of minimal (e.g., < 250 Hz) acoustic information (Gifford et al., 2021; Sheffield and Zeng, 2012; Sheffield et al., 2016, 2015; Sheffield and Gifford, 2014; Zhang et al., 2010). Further, this minimum bandwidth need not include the fundamental frequency (f0) itself and may be centered at a higher frequency to encompass higher harmonics (e.g., Sheffield and Gifford, 2014). Bimodal benefit may continue to grow as acoustic bandwidth increases (e.g., Sheffield and Gifford, 2014) or begin to asymptote at ~500 Hz (D’Onofrio and Gifford, 2021; Gifford et al., 2021; Zhang et al., 2010). Given these observations, bimodal benefit may be driven by a listener’s ability to extract f0 and/or temporal fine structure (TFS) information such as formant peaks from the lowpass or bandpass acoustic signal. Multiple studies have demonstrated the importance of the f0 for speech-in-noise segregation in bimodal listeners and simulations of bimodal hearing (Brown and Bacon, 2009; Chang et al., 2006; Kong et al., 2005; Sheffield and Zeng, 2012), whereas others have suggested that TFS cues may be equally important (Kong and Carlyon, 2007; Li and Loizou, 2008; Sheffield and Gifford, 2014).

The apparent importance of f0 and/or TFS cues in bimodal speech recognition has motivated recent investigations on how bimodal signals are neurally represented and whether encoding of bimodal speech features can be extracted from individual listeners using the frequency following response (FFR; Ananthakrishnan et al., 2016; Anderson et al., 2013; Eng et al., 2022; Xu et al., 2023). The FFR is a scalp-recorded auditory evoked potential primarily originating in the rostral auditory brainstem that reflects neural activity related to transient and sustained portions of speech (Aiken and Picton, 2008; Skoe and Kraus, 2010). An ever-growing body of literature has demonstrated that f0 and formant components of the FFR may both be predictive of speech discrimination in quiet and noise across the lifespan and in listeners with normal or impaired hearing (e.g., Anderson et al., 2013, 2011, 2010b, 2010a; Song et al., 2011). However, use of the FFR to examine how cochlear implant/vocoded or bimodal speech is represented in the auditory system remains sparse. Ananthakrishnan and colleagues (2017) observed robust “envelope” FFRs evoked by vocoded speech stimuli in listeners with normal hearing, demonstrating that amplitude modulations carried by vocoded noise bands or sine waves are sufficient to induce scalp-recorded neural activity at the f0 and its harmonics. The same group observed that FFRs evoked by lowpass filtered speech had reduced f0 amplitudes until the lowpass bandwidth of the stimulus reached or exceeded 750 Hz, demonstrating the importance of both resolved and unresolved harmonics in f0 encoding (Ananthakrishnan et al., 2021). Kessler and colleagues (2020a) evaluated FFR f0 and TFS components evoked from the non-implanted ears of adult bimodal patients. The authors found a significant correlation between FFR f0 amplitude and bimodal benefit, yielding objective evidence that f0 encoding in the non-implanted ear supports perceptual bimodal benefit. Interestingly, some patients in this study demonstrated robust first-formant (F1) encoding (see Kessler et al., 2020a Figure 4), but many listeners had F1 responses at or only slightly above the measurement noise floor, making meaningful relationships between F1 encoding and perceptual bimodal benefit difficult to assess. The lack of F1 representation in a large proportion of patients in this study may be related to the severity (and slope) of hearing loss at 750 Hz, which ranged from mild to profound. Because F1 FFR components have been shown to accurately predict perceptual vowel identification in normal-hearing listeners using unprocessed speech (Svirsky et al., 2017; Won et al., 2016), further inquiry is warranted to determine if this extends to both simulated and true bimodal speech signals. Using the FFR as a tool to objectively assess bimodal speech discrimination may allow clinicians to fine-tune bimodal configurations or provide additional evidence for recommending a second implant.

The goals of this experiment were to:

1. Parametrically examine how f0 and F1 FFR components evoked by simulated bimodal speech are influenced by lowpass acoustic bandwidth manipulations in the ear contralateral to the vocoded signal (i.e., the simulated “non-implanted” ear). We predicted that vocoded-only speech signals would evoke robust f0 components and negligible F1 components. The latter expectation is because cochlear implants and vocoders mainly represent formants as relative intensity differences between electrode pulses in a tonotopic array or as differences across spectral noise bands, respectively. These “rate-place” F1 representations thus do not have a periodic TFS, which is a requirement for evoking FFR F1 components; however, F1 encoding may be improved when additional acoustic input from the non-implanted ear provides complementary formant representation by inducing neural phase locking to periodic TFS components. Consequently, FFR f0 and especially F1 amplitudes were expected to increase as acoustic bandwidth increased in the ear contralateral to the vocoded signal.
2. Assess how objective discrimination of FFRs to different bimodal speech sounds is influenced by lowpass acoustic bandwidth manipulations. We hypothesized that machine learning classification accuracy of FFR spectra evoked by different bimodal speech sounds would improve with increasing acoustic bandwidth in the ear contralateral to the vocoded signal. In this context, machine learning classification provides a method for objectively assessing neural speech discrimination of bimodal signals (Cheng et al., 2021; Smith, 2022; Xie et al., 2019). Our hypothesis was motivated by work suggesting that low-pass acoustic hearing may allow listeners to better discriminate between speech sounds based on their spectral (i.e., formant) contours compared to vocoded or cochlear implant processed speech alone, which biases neural phase-locking to the temporal envelope.
3. Assess whether neural bimodal benefit, measured from the FFR, is predictive of perceptual bimodal benefit on a speech-in-noise task. We first hypothesized that, consistent with previous reports (D’Onofrio and Gifford, 2021; Sheffield et al., 2016; Sheffield and Gifford, 2014), perceptual bimodal performance for adult listeners would improve with increasing acoustic bandwidth in the ear contralateral to the vocoded signal. Further, we hypothesized that improvements in f0 and F1 FFR amplitudes with increasing acoustic bandwidth would predict perceptual bimodal benefit.

To achieve these goals, we measured FFRs and perceptual accuracy to simulated bimodal speech in the following five experimental conditions, in which the vocoded signal was always presented to the right ear, and the lowpass acoustic signals were always presented to the left ear: Vocoder-only, Vocoder +125 Hz, Vocoder +250 Hz, Vocoder +500 Hz, and Vocoder +750 Hz. FFRs were evoked by three naturally-produced vowels (/*ε*/, /i/, and /ʊ/) presented in the five listed bimodal conditions. The original vowels had identical average fundamental frequencies (130 Hz), ensuring that their temporal envelope periodicities were equivalent. We thus expected the FFR spectra evoked by the vocoder-only conditions of the three vowels to be highly similar and less objectively discriminable. Further, we expected that FFR spectra would become more objectively discriminable using a machine learning algorithm due to better neural representation of spectral differences (i.e., F1 peaks) between the vowels when acoustic bandwidth in the ear contralateral to the vocoder increased. In addition to FFR measurements, perceptual performance was also obtained using a modified version of the BKB-SIN test in which test materials were presented in the five bimodal conditions. Predictive relationships between neural and perceptual bimodal benefit were then modeled.

## 2. Materials and methods

### 2.1. Participants

All study procedures were approved by the University of Texas at Austin Institutional Review Board, and all participants provided written consent prior to entering the study. Eighteen adults (12 women) with no history of audiologic or neurologic injury were enrolled. Participants had a mean age of 25.5 years old with a range of 21 to 48 years old. All had normal hearing defined as audiometric thresholds ≤ 25 dB HL from 250 through 8000 Hz bilaterally. Each participant completed five hours of testing for which they were compensated.

### 2.2. Stimuli

**Fig. 1.**
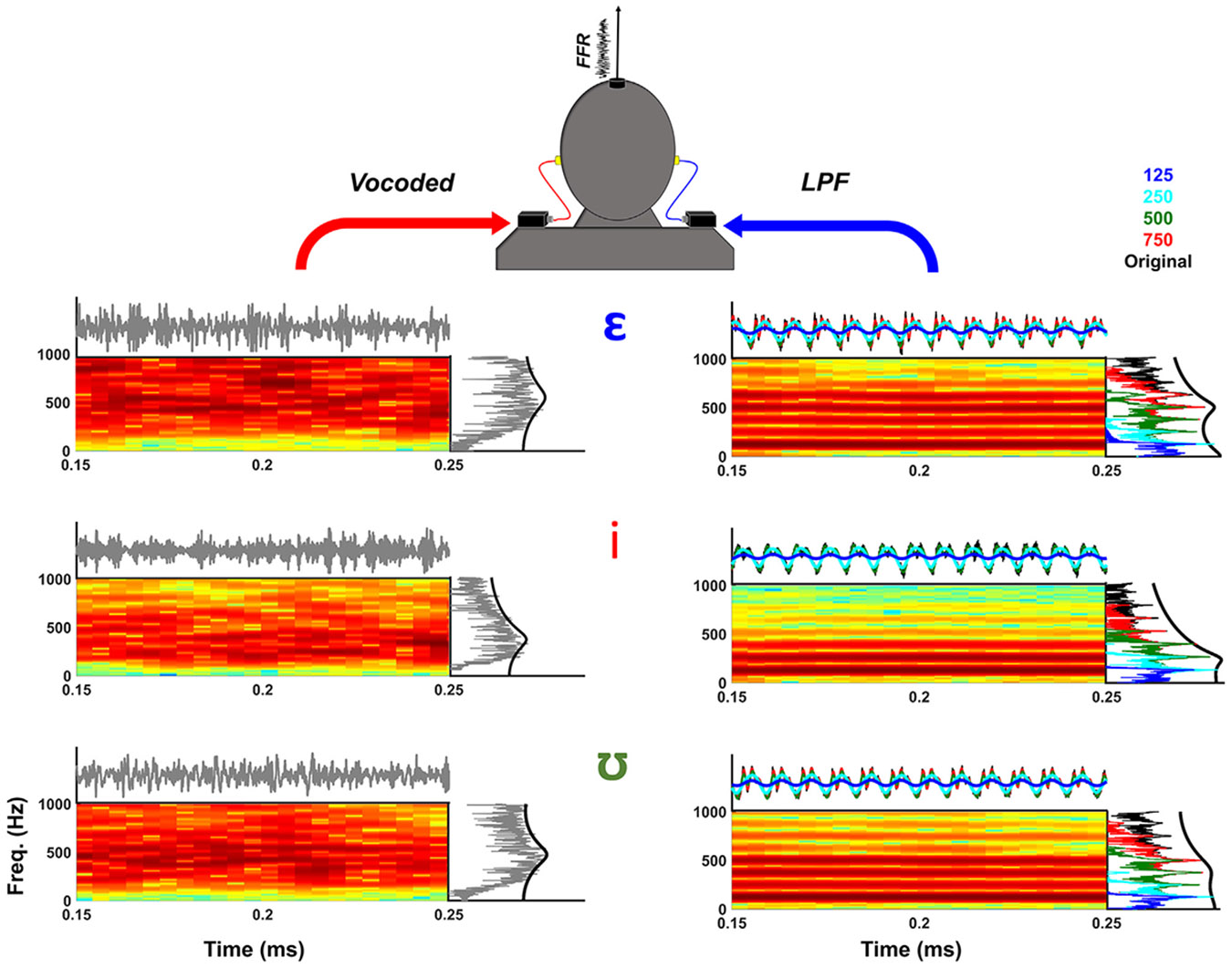
Simulated Bimodal Vowel Stimuli. FFRs were evoked by /*ε*/, /i/, and /ʊ/ vowels in five conditions: Vocoder-only, Vocoder +125 Hz, Vocoder +250 Hz, Vocoder +500 Hz, and Vocoder +750 Hz. Waveforms, spectrograms, and spectra (collapsed over time during the vowel portion of each stimulus) are shown in each panel for vocoded (right ear) and lowpass filtered (left ear) signals. For reference, the original vowel stimulus is shown in black in the waveform and spectra panels for the left ear. Estimated F1 peaks using linear predictive coding are also overlaid on the spectra for vocoded (right ear) and original spectra (left ear) for reference. The spectrograms are from the original stimuli. Lowpass filtered signal waveforms and spectra are color coded using the legend on the upper right of the figure.

All stimuli used in the present study were bimodal simulations of naturally-produced prerecorded speech. The FFR stimuli were first recorded (44,100 Hz sampling rate) from a male speaker producing /hVd/ words with the 12 American English vowels as nuclei (as in Hillenbrand et al., 1995). The speaker was instructed to maintain a constant voice pitch across utterances and produce words of approximately equal length. Three /hVd/ words, /h*ε*d/, /hid/, and /hʊd/, were chosen from this generated corpus due to their relatively low F1 frequencies: 520 Hz, 260 Hz, and 390 Hz, respectively. This consideration is important for FFR measurement, as lower frequencies (< 1000 Hz) generate more robust neural phase locking in the auditory brainstem (Bidelman and Powers, 2018). The length of each word was 380-400 ms long, and the average f0 for each vowel nucleus was 130 Hz. The three naturally produced words were manipulated with a vocoder and by lowpass filtering to create cochlear implant and non-implanted ear simulations, respectively. An eight-channel white noise vocoder, as implemented by AngelSim Software (Fu, 2019), was used to create the cochlear implant simulations. The vocoder used the Greenwood function (Greenwood, 1990) to generate eight tonotopically-spaced carrier filter bandwidths within the 200-7000 Hz range. The low-pass cutoff frequency for envelope detection was set at 400 Hz with a 24 dB/octave roll-off. Non-implanted ear simulations were created by lowpass filtering the original speech samples in MATLAB (The MathWorks Inc., Natick, MA) using an 8^th^ order Butterworth filter (48 dB/octave) with the following cutoff frequencies: 125, 250, 500, and 750 Hz (Figure 1). The decision to use 750 Hz as the maximum cutoff for lowpass filtering was based on previous research indicating that the “average” adult bimodal listener has residual low frequency hearing with the audiometric knee-point at ~750 Hz (Gifford & Dorman, 2019). Vocoder (right ear) and low-pass (left ear) signals were combined after aligning the temporal envelopes of each signal via cross-correlation. This step was taken to ensure that any phase shifts induced by the vocoding process were corrected and that vocoded and lowpass signals were maximally aligned to evoke robust FFRs.

Perceptual testing was completed using a modified version of the BKB-SIN test (Etymotic Research Inc., Elk Grove Village, IL), which contains target sentences presented in four-talker babble at variable signal-to-noise ratios. Target sentences and four-talker babble were each manipulated using the same method described for FFR stimuli to create bimodal BKB-SIN test materials. Five versions of each BKB-SIN list were generated (Vocoder-only, Vocoder +125 Hz, Vocoder +250 Hz, Vocoder +500 Hz, and Vocoder +750 Hz).

### 2.3. FFR acquisition and data reduction

For FFR acquisition, participants were seated in a reclining chair in a double-walled sound booth and were asked to remain still and quiet throughout the experiment. Auditory stimuli were presented in alternating polarity through electromagnetically shielded ER-3 insert earphones (Etymotic Research Inc., Elk Grove Village, IL). A captioned video of each participant’s choosing was played through a Dell PC monitor outside of the sound booth to ensure that participants remained awake but passive. All experiment stimuli were presented in condition-randomized blocks via Neuroscan’s GenTask module (Compumedics Neuroscan, Charlotte, NC). Vocoded speech was always presented at 80 dB SPL in the right ear. The volume of the lowpass signal in the left ear was set such that the level of the original unfiltered stimulus was also 80 dB SPL (Sheffield et al., 2016; Sheffield and Gifford, 2014). The loudness of the lowpass filtered stimuli was not adjusted for each condition despite the fact that the stimuli were likely perceived as softer than the vocoded speech, particularly for lower lowpass frequency cutoffs. This decision was made because the output intensity at each individual frequency would vary across bandwidth conditions if stimuli were adjusted to have equal loudness, which would confound FFR measurements, BKB-SIN performance, and bimodal benefits comparisons (Sheffield et al., 2016; Sheffield and Gifford, 2014). Each stimulus was presented in each polarity (“A” and “B”) 1000 times.

FFRs were recorded on a Neuroscan SynAmps2 system (Compumedics Neuroscan, Charlotte, NC) using a 10,000 Hz sampling rate. Testing was completed using a single-channel bipolar montage: Fpz (+), linked mastoids (−), and forehead (GND). Continuous data were exported from Curry 8 software and analyzed offline in MATLAB (The MathWorks Inc., Natick, MA). All continuous data were first bandpass filtered from 70-1000 Hz and epoched from – 50 - 500 ms relative to stimulus onset. Each epoch was detrended, artifact rejected at ± 70 μV, and baseline corrected using the pre-stimulus (−50 - 0 ms) interval.

#### 2.3.1. FFR Spectra

FFR spectra were analyzed to address effects of bimodal bandwidth on f0 and F1 amplitudes (Goal 1) and for objective discrimination using machine learning classification (Goal 2). To create FFR spectra, cleaned single-trial epochs were first grouped and averaged together by condition, creating grand average FFR waveforms for each stimulus in each polarity (“A” and “B”). Previous studies have shown that adding together FFR waveforms obtained with each polarity ((A+B)/2) yields responses that accentuate the stimulus envelope (f0) while attenuating TFS components (Aiken and Picton, 2008). Conversely, subtracting one response polarity from the other ((A-B)/2) attenuates the envelope and accentuates TFS. Because we wanted our FFR analyses to capture both f0 (envelope) and F1 (TFS) components from each stimulus within the same measurement, we used FFR spectra evoked by a single polarity (polarity A) in our spectral analyses (Krizman and Kraus, 2019; Yellamsetty and Bidelman, 2019). This decision was made during pilot testing by comparing added, subtracted, and single polarity FFR spectra evoked by the original unprocessed stimuli to the spectra of those stimuli. FFR spectra from the steady-state vowel portions (150-250 ms) of each averaged “polarity A” FFR waveform were extracted using fast-Fourier transforms. Each spectrum was comprised of 250 points within the 0-1000 Hz range (4 Hz spectral bin width).

#### 2.3.2. FFR phase-locking value spectra

**Fig. 2.**
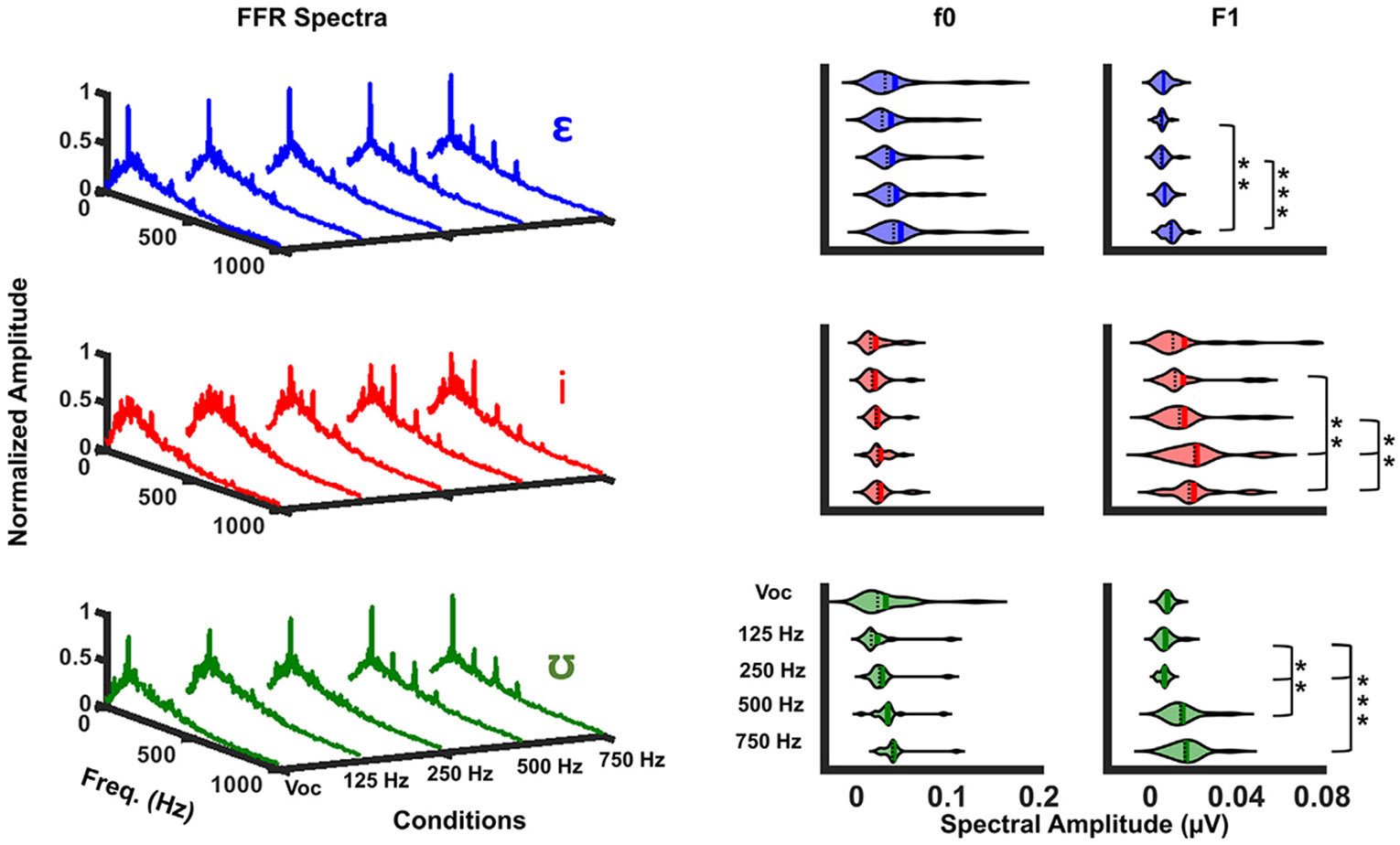
Bimodal FFR Spectra. FFR spectra for each vowel and condition are shown in the left column. Violin plots in the right column show kernel density distributions of f0 and F1 spectral amplitudes for each vowel and condition (black dotted lines = mean, solid lines = median). *p < .05, **p < .01, ***p < .0001.

The FFR spectra described above provide a convenient method for assessing f0 and F1 component amplitudes extracted from grand average waveforms of multiple trials. This type of analysis was chosen because it is computationally straightforward and could be conducted on most commercially-available auditory evoked potentials systems. However, FFR spectral analysis is negatively impacted by two factors. The first is that the spectral noise floor varies as a function of frequency, such that residual noise amplitude is given approximately as 1/frequency (Buzsáki, 2006; Zhu et al., 2013). The second is that the amplitudes of FFR spectral components decay and approach the noise floor as frequency increases due to phase-locking limitations of the auditory brainstem (Bidelman and Powers, 2018). The combined influences of each factor can be seen in the FFR spectra in Fig. 2.

Because of the issues described above, we used a more computationally intensive analysis of the FFR, the phase locking value (PLV) spectrum, when modeling predictive relationships between neural and behavioral bimodal benefit (Goal 3). The PLV is a number between 0 and 1 reflecting trial-by-trial phase coherence of the FFR within specified time-frequency bins (Dobie and Wilson, 1989; Zhu et al., 2013); a PLV of 0 indicates no phase coherence within a time-frequency bin, whereas a value of 1 indicates complete phase coherence. When PLVs are averaged across a time range of interest, the outcome is a PLV spectrum, which depicts average phase-locking strength as a function of frequency. One advantage of using the PLV spectra over traditional spectra is that the PLV is an “amplitude-less” measure and is therefore not as impacted by the spectral analysis issues described above. A disadvantage of using the PLV is that it is less easily implemented on commercial/clinical auditory evoked potentials equipment, as computations are performed on a trial-by-trial basis as opposed to grand average waveforms.

PLVs were obtained using an approach similar to Zhu and colleagues (Zhu et al., 2013). First, each cleaned single-trial FFR epoch was short-time fast Fourier transformed using zero-padded 60 ms sliding windows with 1-ms overlap. This resulted in single-trial spectrograms with 1 ms temporal resolution and (interpolated) 1 Hz frequency resolution. Response magnitudes at each time-frequency point were normalized to a value of 1. Response phases were extracted from each time-frequency point in each single-trial spectrogram. For each response polarity (“A” and “B”), 1000 vectors were summed at each time-frequency point to generate a PLV. PLVs were averaged across time from the 150-250 ms steady-state portion of the FFR, resulting in a PLV spectrum for each polarity. The PLV spectra for each polarity were then averaged together. Phase locking strength for each of the three vowels in each of the five conditions were obtained at f0 (130 Hz) and F1 frequencies (/*ε*/ = 520 Hz, /i/ = 260 Hz, and /ʊ/ = 390 Hz). f0 and F1 PLVs were averaged across the three vowels for each bimodal condition, resulting in single PLVs that captured f0 and F1 phase-locking strength across stimuli. Estimates of the PLV noise floor at each time-frequency point were also obtained by analyzing the pre-stimulus EEG (−50-0 ms) in the same manner described above. This approach ensured that the PLVs measured at frequencies of interest were “true” neurophysiologic responses above the empirical noise floor.

### 2.4. Bimodal BKB-SIN test procedure

Each BKB-SIN list is divided into two list pairs (“A” and “B”). The starting SNR of the first sentence in each list pair is +21 dB and decreases in 3 dB steps with each subsequent sentence down to 0 dB. The listener is instructed to repeat target sentences to the tester while ignoring babble noise, and the tester scores the number of key words correct at each SNR for each list pair. The number of correct key words is used to quantify the dB SNR at which the listener’s performance approaches 50% for each list pair; dB SNRs from both list pairs are averaged to yield a dB SNR threshold.

Due to limitations imposed by the COVID-19 pandemic, bimodal BKB-SIN testing was done remotely by presenting stimuli through a Qualtrics survey (Qualtrics, Provo, UT) and recording spoken responses through integrated Phonic software (Phonic, Sunnyvale, CA). The recorded responses were used to score each participant’s performance. All participants wore earbuds and were instructed to set the volume to “comfortable conversation level” while listening to an unmanipulated practice list at the beginning of the survey. They were instructed not to change the volume for the remainder of the test. The order in which each condition was presented was randomized across participants.

### 2.5. Statistical analyses

#### 2.5.1. Bimodal bandwidth effects on FFR spectral amplitudes and perceptual performance

Repeated measures analysis of variance was conducted to determine the effects of bimodal condition (Vocoder-only, Vocoder +125 Hz, Vocoder +250 Hz, Vocoder +500 Hz, and Vocoder +750 Hz), vowel type (/*ε*/, /i/, and /ʊ/), and spectral component (f0 and F1) on FFR spectral amplitudes (SPSS version 25). Similarly, effects of bimodal condition on BKB-SIN dB SNR thresholds were also assessed. Post-hoc pairwise comparisons with Bonferroni adjustment were used to evaluate which levels within factors were significantly different in cases where main effects were observed.

#### 2.5.2. Machine learning classification of bimodal FFR spectra

Machine learning algorithms have been used previously to assess whether the information contained in FFR waveforms or spectra is sufficient to decode the stimulus classes that evoked them (Cheng et al., 2021; Llanos et al., 2017; Sadeghian et al., 2015; Smith, 2022; Xie et al., 2018, 2019; Yi et al., 2017). In this approach, FFR classification accuracy (i.e., how well FFRs are correctly labeled by the machine learning algorithm) serves as an objective measure of stimulus discrimination. FFR classification accuracy can be compared between levels of an independent variable (e.g., when measured pre- vs. post auditory training or during passive vs. active listening) to determine how these factors impact classification performance (e.g., Cheng et al., 2021; Xie et al., 2018). In the present study, we expected vowel identity to be better represented in bimodal FFRs as acoustic bandwidth in the non-implanted ear increased; thus, we predicted that machine learning classification accuracy of bimodal FFRs would improve as stimulus bandwidth increased.

A linear support vector machine (SVM; Cristianini and Shawe-Taylor, 2000) was created in MATLAB following the general procedures described by Xie and colleagues (2019). The linear SVM input features were the FFR spectra (described above) expressed in principal component feature space in order to reduce the size of the input to the model. The top five principal components, which captured 75% of the variance in FFR spectra, were used. Linear SVM model outputs were vowel type (/*ε*/, /i/, and /ʊ/). Note that standard linear SVM can only classify data into binary classes; thus a “one-against-one” strategy was used in which the linear SVM constructed N(N-1)/2 classifiers (N=3 in this experiment, as three vowels were used). After FFR classification was performed on every possible pairwise combination, the class with the highest accuracy was used as the classification label.

The linear SVM model was cross-validated using a three-fold approach iterated 1000 times for each of the five bimodal conditions (see Xie et al., 2019, Figure 1). Within each bimodal condition, each iteration of the linear SVM classifier used 18 FFR spectra (one from each participant). The spectra were randomly and equally divided into 3 groups (or folds) containing 6 participants. A “leave-one-out” strategy then utilized two of the three folds to train the classifier. After the classifier was trained, the held-out fold was then used as test-case data. This procedure was repeated within each iteration such that each fold was held-out as the test-case data, whereas the remaining two folds were used for training the classifier. For each iteration, average classifier accuracy across cross-validations was calculated. Outcomes of the 1000 model iterations were also used to create grand total cross-validation accuracies as well as a bootstrapped distribution of classifier accuracies for each of the five bimodal conditions. A null distribution of model accuracies was also generated using Vocoder + 750 Hz condition dataset. The null (or “chance”) distribution was created using the steps described above, except that model outputs (i.e., the “true” stimulus labels /*ε*/, /i/, and /ʊ/) were randomly assigned to bimodal FFR spectra inputs on each iteration of the loop. The statistical significance of each classifier’s performance was determined using p=(a+1)∕(n+1), where a indicates the number of observations from the null classification distribution that surpasses the median of the “true” distribution and n is the number of observations comprising the null distribution (Phipson and Smyth, 2010). This equation was used to determine if FFR spectra from each bimodal condition were classified with accuracies above the empirical null distribution and to test whether classification accuracy distributions were significantly different between the five bimodal stimulus conditions.

#### 2.5.3. Predictive relationships between neural and perceptual bimodal benefit

The third goal of this study was to assess whether neural bimodal benefit was predictive of perceptual bimodal benefit. We derived neural bimodal benefit at f0 and F1 by calculating the across-vowel averaged PLV difference between the Vocoder-only and Vocoder + 750 Hz conditions. The outcome of this calculation provided a metric for neural phase locking improvements at f0 and F1 when going from the most spectrally-sparse stimulus (Vocoder-only) to the most spectrally-rich stimulus (Vocoder + 750 Hz). BKB-SIN bimodal benefit was obtained by calculating the difference in dB SNR threshold between Vocoder-only and Vocoder + 750 Hz conditions. Neural bimodal benefit at f0 and F1 were used as independent variables and perceptual bimodal benefit was used as a dependent variable in a linear regression analysis (SPSS version 25).

## 3. Results

### 3.1. Bimodal FFR spectral amplitudes as a function of bandwidth

A repeated measures ANOVA was conducted to determine the effects of bimodal condition (Vocoder-only, Vocoder +125 Hz, Vocoder +250 Hz, Vocoder +500 Hz, and Vocoder +750 Hz), vowel type (/*ε*/, /i/, and /ʊ/), and spectral component (f0 and F1) on FFR spectral amplitudes.

Degrees of freedom were corrected using Greenhouse-Geisser estimates of sphericity due to a violation of the assumption of sphericity. Though there was no significant main effect of vowel type [F(1.25, 21.24) = 1.78, p = 0.2, eta2[g] = 0.09], results showed significant main effects of bimodal condition [F(1.60, 27.21) = 13.83, p < 0.001, eta2[g] = 0.45], and spectral component [F(1.00, 17.00) = 29.40, p < 0.0001, eta2[g] = 0.63] on FFR spectral amplitude. Significant interaction effects were found between bimodal condition and vowel type [F(4.24, 72.11) = 4.76, p < 0.01, eta2[g] = 0.22], and spectral component and vowel type [F(1.76, 29.89) = 12.9, p < 0.0001, eta2[g] = 0.43], showing the effects of bimodal condition or spectral component may depend on the vowel type. Results showed no significant interaction effects between bimodal condition and spectral components or the three independent variables at the 0.05 level, indicating the effect of these factors did not depend on each other.

Post-hoc analyses with Bonferroni adjustment for multiple comparisons were conducted to examine which pairs of comparison between levels of factors drove the statistical significance. The multiple comparisons between vowel type and bimodal condition indicated that the FFR spectral amplitudes to /*ε*/ were significantly higher in Vocoder +750 Hz than Vocoder +125 Hz [0.007 (95%CI, 0.001 to 0.007) *μ*V, p < 0.01] and Vocoder +250 Hz [0.006 (95%CI, 0.003 to 0.012) *μ*V, p < 0.0001]. Additionally, the FFR amplitudes to /i/ were significantly higher in Vocoder + 750 Hz than Vocoder + 125 Hz [0.005 (95%CI, 0.001 to 0.009) *μ*V, p < 0.01] and Vocoder + 250 Hz [0.004 (95%CI, 0.001 to 0.007) *μ*V, p < 0.01], and were significantly higher in Vocoder +500 Hz than Vocoder +125 Hz [0.006 (95%CI, 0.002 to 0.01) *μ*V, p < 0.01] and Vocoder +250 Hz [0.004 (95%CI, 0.001 to 0.008) *μ*V, p < 0.01]. FFR amplitudes to /ʊ/ were also significantly higher in Vocoder +750 Hz than Vocoder +125 Hz [0.014 (95%CI, 0.009 to 0.019) *μ*V, p < 0.0001], Vocoder +250 Hz [0.012 (95%CI, 0.008 to 0.015) *μ*V, p < 0.0001], and Vocoder +500 Hz [0.004 (95%CI, 0.001 to 0.007) *μ*V, p < 0.05]. Moreover, FFR amplitudes to /ʊ/ were significantly higher in Vocoder +500 Hz than Vocoder +125 Hz [0.01 (95%CI, 0.004 to 0.015) *μ*V, p < 0.0001] and Vocoder +250 Hz [0.008 (95%CI, 0.003 to 0.012) *μ*V, p < 0.05].

The multiple comparisons between vowel type and spectral component suggested that FFR amplitudes to f0 were significantly higher than amplitudes to F1 for vowels /*ε*/ [0.029 (95%CI, 0.017 to 0.041) *μ*V, p < 0.0001] and /ʊ/ [0.021 (95%CI, 0.01 to 0.032) *μ*V, p < 0.01], whereas there was no significant difference between FFR spectral amplitudes in two spectral components for vowel /i/.

In general, these results were consistent with the spectral amplitudes of the original stimuli and the expectation that F1 components were better represented in the FFR after the lowpass cutoff in the simulated nonimplanted ear surpassed the F1 frequency for each vowel.

### 3.2. Machine learning classification of bimodal FFR spectra

**Fig. 3.**
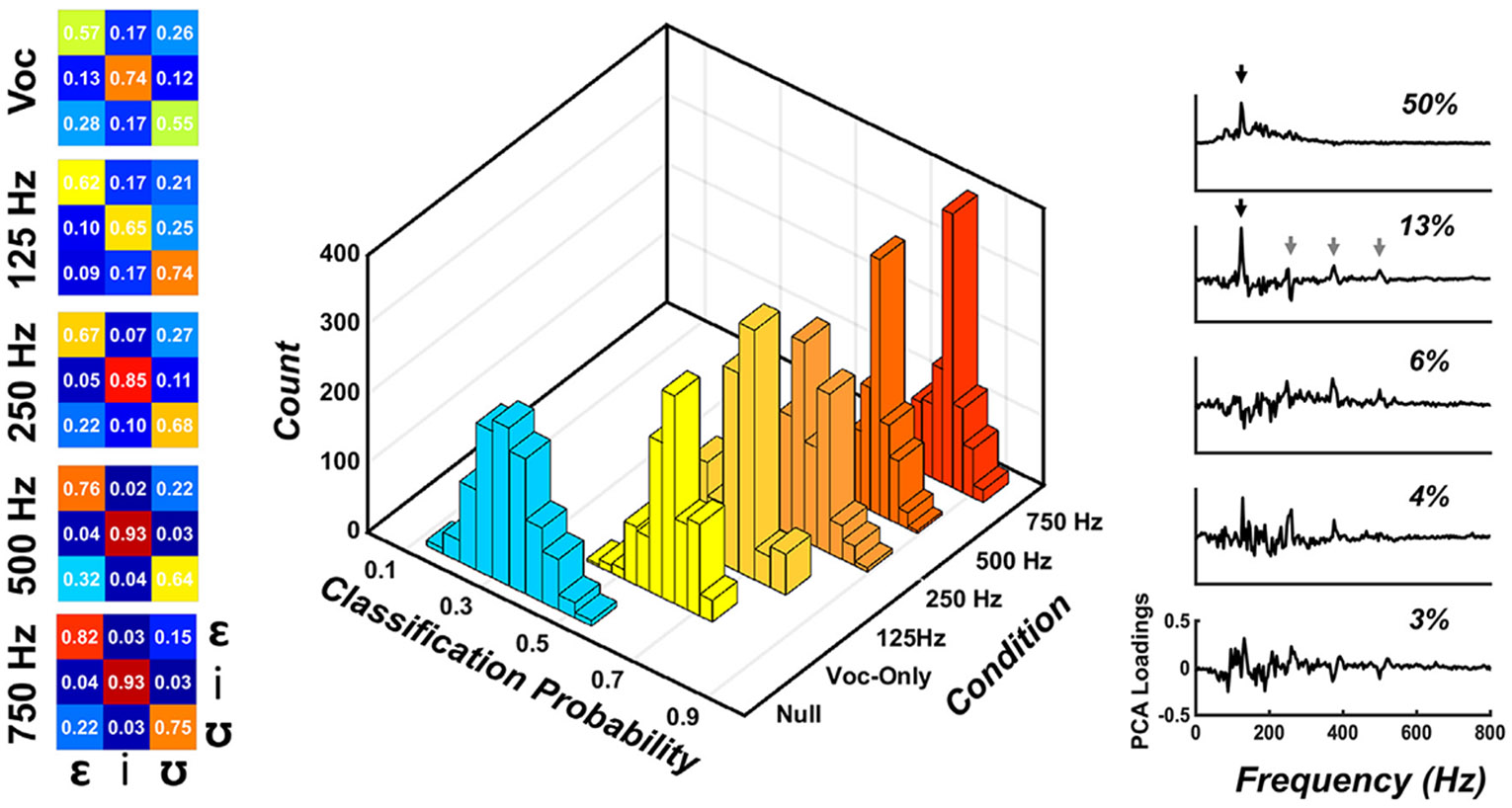
Objective Discrimination of Bimodal Vowel FFRs. Confusion matrices produced by averaging 1000 iterations of the linear SVM model for each bimodal condition are shown on the left. Note that Voc represents right monaural presentation of the vocoded stimulus, whereas the other bimodal conditions are binaural. The 3D histogram in the middle depicts model accuracy distributions for each bimodal condition over 1000 iterations as well as a null (“chance”) distribution. Principal components analysis (PCA) loadings for the Vocoder +750 Hz condition (right) demonstrate that the model used both f0 (black arrow) and F1 (gray arrows) features to perform classification.

Figure 3 depicts linear SVM classification accuracies over 1000 iterations of the model for each bimodal condition. The confusion matrices on the left display grand average classification accuracies for each bimodal condition and vowel. The 3D histograms in the center represent overall classification accuracy distributions for all 1000 iterations of the model for each bimodal condition, as well as the empirical null distribution generated by randomly shuffling classifier outputs (i.e., response labels) for each iteration. Classification accuracy distributions for each condition were significantly above the empirical null distribution (p < 0.001). The classification accuracy distribution for each bimodal condition was not significantly different from its immediate neighbor (e.g., Vocoder-only was not different from Vocoder + 125 Hz, etc.); however, all other comparisons did significantly differ (by at least p < 0.05). These results indicate a systematic improvement in FFR classification as bimodal bandwidth increased from Vocoder-only to Vocoder + 750 Hz. The right panel of Figure 3 demonstrates the first five principal components (explaining 75% of variance) that were extracted from the Vocoder + 750 Hz FFR spectra. These plots indicate the most salient features of the FFR spectra that were used by the model to perform classifications were the f0 (black arrow) and F1 frequencies of each vowel (/*ε*/ = 520 Hz, /i/ = 260 Hz, and /ʊ/ = 390 Hz; gray arrows).

### 3.3. BKB-SIN performance as a function of bandwidth

**Fig. 4.**
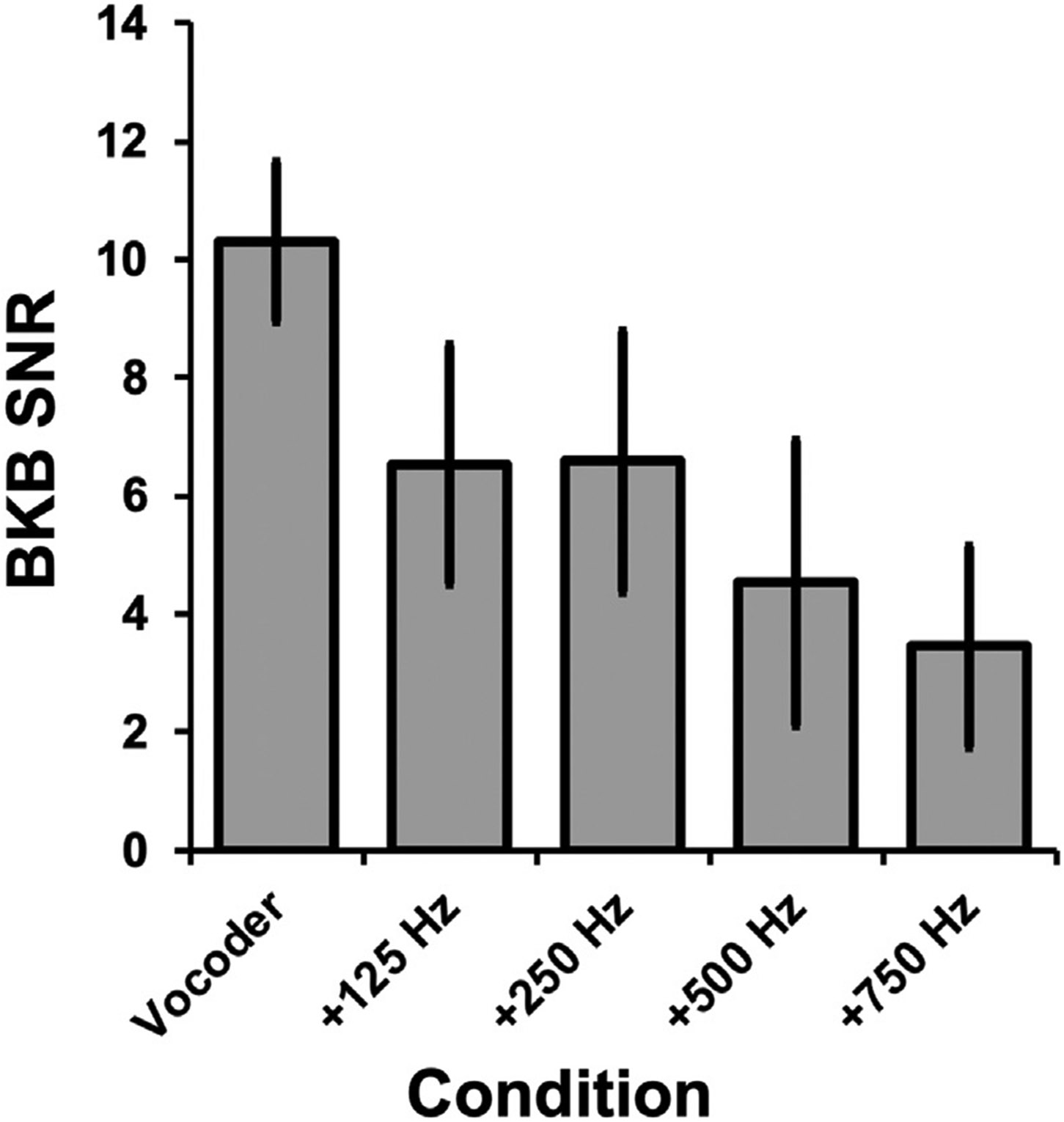
BKB Sentence Performance as a Function of Bimodal Bandwidth. Average BKB SNRs decreased (“improved”) as bimodal bandwidth increased. Error bars = ± 1 S.D.

As a general trend, BKB-SIN SNR losses were higher (“poorer”) for signals with less contralateral acoustic information and became smaller (“better”) as more contralateral acoustic content became available (Fig. 4). A repeated measures analysis of variance (ANOVA) revealed that BKB-SIN performance differed significantly across bimodal stimulus conditions [F(4, 68) = 44.84, p < 0.0001, eta2[g] = 0.73]. Post-hoc analyses with Bonferroni adjustment for multiple comparisons revealed that all pairwise differences between bimodal stimulus conditions were statistically significant except the comparisons between Vocoder + 125 Hz and Vocoder + 250 Hz, Vocoder + 125 Hz and Vocoder + 500 Hz, and Vocoder + 500 Hz and Vocoder + 750 Hz. Thus, mean BKB-SIN SNR losses in the Vocoder-only condition were significantly higher than all other conditions. Additionally, SNR losses in the Vocoder + 250 Hz condition were significantly higher than Vocoder + 500 Hz and Vocoder + 750 Hz conditions.

### 3.4. Predictive relationships between physiologic and perceptual bimodal benefit

As discussed above, PLV spectra were calculated from the raw trial-by-trial FFR data for each bimodal condition and vowel. PLV spectra differences between Vocoder-only and Vocoder + 750 Hz conditions were calculated as neurophysiologic metrics of bimodal benefit at f0 and F1 frequencies. The across-vowel average PLV bimodal benefits at f0 and F1 frequencies were used to predictively model perceptual bimodal benefit on the BKB Sentence test using multiple linear regression. Before running the regression model, the PLV bimodal benefits were first transformed to Fisher’s z scores to create standardized normal distribution of the variables. Multicollinearity was also checked by computing the Variance Inflation Factor (VIF) for each independent variable in order to measure the strength of correlation between two predictors, f0 and F1. The VIF value for the two predictors was 2.71, which indicated a moderate correlation between the two predictors. Because the purpose of the model was to determine the combined predictive power of f0 and F1 on perceptual performance, both predictors were initially included despite the correlation between them. The results suggested that the model with two predictors explained 37% of the variance (R^2^ = .37), and the two variables (f0 and F1 PLV) significantly predicted perceptual bimodal benefits [F(2, 15) = 4.44, p < 0.05]. However, neither f0 nor F1 PLV individually had a significant prediction on perceptual bimodal benefits (both p > 0.05), which may be due to the correlation between these two predictors. These results indicated that this model had a significant predictive power of two predictors as a whole on the perceptual bimodal benefits; however, this model did not conclude the significance of f0 PLV and F1 PLV individually.

Two individual linear regression analyses were also run to predict the perceptual bimodal benefits from f0 PLV and F1 PLV, respectively. The model with f0 PLV as a predictor explained 33% of the variance (R^2^ = .33), and f0 PLV significantly predicted perceptual bimodal benefits [F(1, 16) = 7.78, p < 0.05]. The model with F1 PLV as a predictor explained 34% of the variance (R^2^ = .34), and F1 PLV significantly predicted perceptual bimodal benefits [F(1, 16) = 8.21, p < 0.05].

## 4. Discussion

### 4.1. Bimodal FFR spectral amplitudes as a function of bandwidth

The first goal of this study was to parametrically examine how f0 and F1 FFR components evoked by simulated cochlear implant-alone and bimodal vowels were influenced by lowpass acoustic bandwidth manipulations in the ear contralateral to the vocoded signal. Our analyses revealed that FFR f0 was robustly represented in the Vocoder-only conditions for /*ε*/ and /ʊ/ and grew with the addition of lowpass acoustic energy in the contralateral ear. This was likely because increasing bandwidth introduced more resolved harmonics, which modestly bolstered FFR f0 spectral amplitude (Ananthakrishnan et al., 2021). In contrast, the vowel /i/ did not evoke a robust FFR f0 component in the Vocoder-only condition, presumably because the vocoded waveform envelope was dominated by the relatively low F1 frequency (260 Hz); f0 representation for /i/ became modestly evident only when multiple harmonics were accessible through the lowpass acoustic signal in the opposite ear. Taken together, these results support previous perceptual findings suggesting that the addition of acoustic information, even in the form of the first harmonic in isolation, bolsters bimodal benefit by enhancing neural representation of f0 (Brown and Bacon, 2009; Sheffield et al., 2016, 2016; Zhang et al., 2010).

**Fig. 5.**
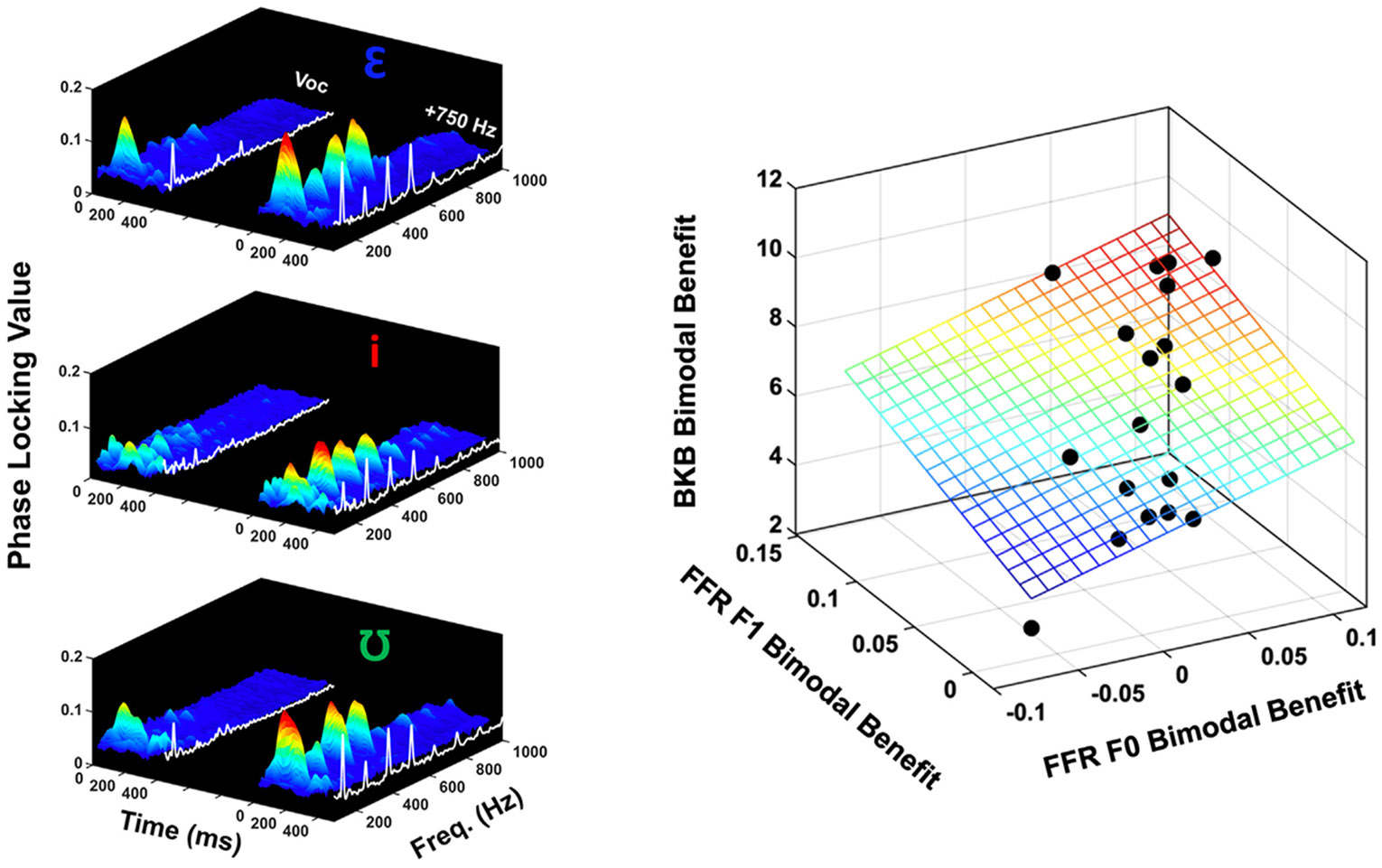
Physiologic Bimodal Benefit Predicts Perceptual Bimodal Benefit. PLV spectrograms and spectra (averaged over the 150-250 ms steady-state vowel portion) for the Vocoder-only (Voc) and Vocoder +750 Hz conditions are shown in the left column. While the Vocoder-only PLV spectra are sparse, the Vocoder +750 Hz responses show phase locking at f0 and multiple harmonics. Notably, F1 frequencies (/*ε*/ = 520 Hz, /i/ = 260 Hz, and /ʊ/ = 390 Hz) and differences in spectral shape related to vowel identity are evident in each PLV spectra for the Vocoder +750 Hz condition. The 3D scatterplot on the right plots BKB SIN bimodal benefit as a function of f0 and F1 bimodal benefit extracted from the PLV spectra for each subject. The mesh grid indicates the plane of predicted values from the regression model.

We predicted that F1 components would be negligible in the Vocoder-only FFR spectra and would grow systematically when the lowpass filter of the acoustic signal increased to encompass the F1 frequency for each vowel. The former prediction is based on the idea that cochlear implants and vocoders mainly represent formants as relative intensity differences between electrode pulses in a tonotopic array or as differences across spectral noise bands, respectively. These “rate-place” F1 representations thus do not have a periodic TFS, which is a requirement for evoking FFR fine structure components. This prediction was incorrect. It can be seen in the spectra (Fig. 2) and PLV spectra (Fig. 5) that small F1 components are present, particularly for /*ε*/ and /i/ vowels, even in the Vocoder-only condition. Because there is no periodic “carrier” fine structure in the vocoded signals, this suggests that the vocoder captured F1 influences on the temporal envelope in addition to the f0. Indeed, the vocoder’s lowpass frequency cutoff for envelope detection was 400 Hz with a 24 dB/octave roll-off; this would have allowed all formants from the vowels in this study to be (at least partially) represented in the temporal envelope and thus induce neural phase locking at F1 in addition to f0. More parametric work examining how vocoder temporal envelope cutoffs and number of channels influence FFR features is warranted (Ananthakrishnan and Luo, 2022).

Although F1 components were present in Vocoded-only FFR spectra, the amplitudes of these components grew significantly when the acoustic lowpass cutoff frequency exceeded the F1 frequency (Fig. 2). This indicates that a major advantage of extending acoustic bandwidth in the non-implanted ear is better neural representation of F1 components. While several perceptual investigations of bimodal hearing have hypothesized this (Kong and Carlyon, 2007; Li and Loizou, 2008; Sheffield and Gifford, 2014), to our knowledge, the present paper is the first to demonstrate it neurophsyiologically in simulated bimodal listeners.

### 4.2. Objective discrimination of bimodal FFRs as a function of bandwidth

The second goal of this study was to assess how objective discrimination of bimodal vowel FFRs is influenced by lowpass acoustic bandwidth manipulations. We hypothesized that machine learning classification accuracy would improve with increasing acoustic bandwidth due to the F1 differences between stimuli being better represented. Our results demonstrated that objective discrimination did improve as bimodal bandwidth increased and that this improvement was related to both FFR f0 and F1 spectral features. While the f0 of each bimodal vowel was the same (130 Hz), vocoder and acoustic waveform envelopes differed (see Fig. 1) because each stimulus has different noise band and harmonic amplitudes that sum together idiosyncratically. FFR f0 amplitudes reflected these idiosyncrasies across vowels, and the machine learning model used f0 as one feature to accurately perform classification. The starkest example of this is the linear SVM’s classification performance on the Vocoded-only responses. Because of the similarities in their f0 amplitudes, the classifier confused /*ε*/ and /ʊ/ for each other approximately 25% of the time, resulting in classification accuracies of 57% and 55%, respectively, for these two vowels. In contrast, the FFR spectra to Vocoded-only /i/ had a small f0 and a more dominant F1 component (260 Hz) in the spectrum. This difference between /i/ and the other two vowel spectra resulted in 74% classification accuracy and fewer confusions. As acoustic bandwidth increased, the classifier began to use F1 information also, as shown in the PCA weights for the Vocoder +750 Hz condition in Fig. 3. These results indicate that even though the contour differences in the original vowel stimuli spectra undergo morphological transformations when they are represented as FFR spectra (described above), the FFR spectra contain sufficient contour differences to increasingly facilitate objective discrimination as acoustic bandwidth increases. While previous studies have demonstrated that FFRs can be used to objectively discriminate a variety of speech features (see Smith (2022) for review), this is the first study to do so with simulated bimodal speech. If this or a similar approach yields comparable results in real bimodal listeners, objective discrimination of FFRs may be a useful clinical tool for fine-tuning bimodal configurations or recommending a second implant.

### 4.3. Using neural bimodal benefit to predict perceptual bimodal benefit

The third goal of this study was to assess whether neural bimodal benefit, measured from FFR f0 and F1 PLV spectral components, is predictive of perceptual bimodal benefit on a speech-in-noise task. This goal was motivated by previous work in normal hearing listeners using unprocessed speech demonstrating that: 1) FFR f0 and F1 amplitudes are variable across normal hearing listeners (Anderson et al., 2012; Bidelman et al., 2014; Clinard et al., 2010; Clinard and Tremblay, 2013; Ruggles et al., 2011; Sadeghian et al., 2015) and 2) predictive relationships exist between FFR f0 and/or F1 encoding and a variety of speech perception tasks across the lifespan (e.g., Anderson et al., 2011, 2010a, 2010b; Song et al., 2011; Svirsky et al., 2017; Won et al., 2016). We extended these findings to hypothesize that bimodal benefits in f0 and F1 encoding are predictive of perceptual bimodal benefits on a speech-in-noise perception task (Kessler et al., 2020a).

Our finding that speech perception improved as bimodal acoustic bandwidth increased was generally consistent with previous work with adult listeners (D’Onofrio and Gifford, 2021; Sheffield et al., 2016; Sheffield and Gifford, 2014), although the magnitude of improvement for each consecutive increase in bandwidth differed. This discrepancy may be related to differences in lowpass filter roll-offs for acoustic stimuli processing between studies and/or differences in test materials used (e.g., consonant-nucleus-consonant words vs. BKB sentences). We built a regression model in which neural representations of f0 and F1 bimodal benefit were used to predict perceptual bimodal benefit. The results of this regression model indicated that 37% of perceptual bimodal benefit variance was accounted for by FFR f0 and F1 bimodal benefit. An important caveat to consider when interpreting our model is the observed multicollinearity between f0 and F1 bimodal benefit: participants with larger f0 bimodal benefit were also more likely to have larger F1 bimodal benefit and vice versa. Multicollinearity can make it difficult to tease apart the coefficients related to each independent variable, as a unit change in one variable is concomitant with a change in the other (Alin, 2010). However, our analysis of the variance inflation factor (VIF) indicated a moderate relationship between the two predictors; in this case, model predictions are considered valid (i.e., BKB bimodal benefit can be predicted using f0 and F1 combined) without correcting for multicollinearity (Neter et al., 1996). Together, our observation that FFR f0 and F1 collectively predicted bimodal benefit is a novel finding. Future work in which FFRs to bimodal signals are measured in the presence of background noise may shed light on whether amplitude dips in babble noise allow the auditory nervous system to “glimpse” f0 and F1 features (e.g., Li and Loizou, 2008).

## 5. Conclusions, limitations, and future work

This study demonstrated that neural representations of f0 and F1 are enhanced with increasing acoustic bandwidths for simulated bimodal signals. The FFR spectral differences that emerge with increasing bandwidth more clearly reveal vowel identity such that FFRs evoked by different vowels can be objectively discriminated with a greater level of accuracy. Lastly, the improvements in f0 and F1 neural encoding with increasing bandwidth are collectively predictive of perceptual bimodal benefit on a speech-in-noise task.

While this study holds promise for extending similar methods to real bimodal patients, some limitations should be considered. The first limitation pertains to simulating the non-implanted ear input for bimodal listeners. Our lowpass filter method for simulating how residual low frequency hearing may function in a real bimodal setup may only be a gross approximation of audibility in the non-implanted ear. Our decision to simulate the non-implanted ear in this way was based on previous perceptual work with similar goals to the present study (e.g., Sheffield et al., 2016). Our stimuli thus do not reflect aspects of hearing aid processing (frequency-specific amplification, compression, and compression attack and release settings) that bimodal listeners would experience. Further, we did not process the non-implanted ear stimuli through a sensorineural hearing loss simulator. Sensorineural hearing loss is known to widen auditory filters by up to four times the width of auditory filters in a normally functioning cochlea (Moore et al., 1999). Wider auditory filters allow more harmonics to interact within the same filter and may explain why FFR f0 encoding is enhanced in listeners with high frequency sensorineural hearing loss (Anderson et al., 2013). However, a similar finding may not be expected for bimodal listeners, as the average bimodal patient has residual hearing in the frequency range where most harmonics would be resolved, even with wider filters. Making bimodal simulations more realistic for future experiments is a goal of our collaborative research team.

A second limitation pertains to FFR measurement in real bimodal listeners. Unlike vocoded speech, cochlear implant-processed speech is relayed to the spiral ganglia electrically. Steady-state electrical pulses can interfere with EEG recordings, as the electric artifact produced by cochlear implant electrode arrays may be orders of magnitude larger than the auditory evoked potential of interest (Deprez et al., 2017; Gransier et al., 2020, 2016; Hofmann and Wouters, 2012). This issue poses a significant challenge when recording FFRs in cochlear implant users. However, possible solutions (e.g., interleaving EEG sampling points between electrical pulses or using independent components analysis to remove artifact and isolate neural activity) have been described elsewhere (Carlyon et al., 2021; Gransier et al., 2021; Intartaglia et al., 2022). It may also be informative to use the FFR to measure neural encoding of acoustic-only information relayed through residual low frequency hearing as reported by Kessler and colleagues (2020a). Another factor that may complicate bimodal FFR recordings is abnormal binaural fusion or temporal misalignment between cochlear implant and hearing aid inputs (e.g., Reiss et al., 2018). In addition to causing perceptual interference, abnormal binaural fusion would misalign neural tracking of both the cochlear implant and acoustic signal. Binaural phase misalignment of dichotic signals has been shown to attenuate the FFR through volume conduction of the monaural phase-locked signals in normal hearing listeners (Gnanateja and Maruthy, 2019; So and Smith, 2021). In the present study, vocoded and lowpass signals were time-aligned and thus reflected the best-case scenario for a bimodal configuration. Given its temporal precision, however, future work is warranted to determine if the FFR is a useful tool for aligning binaural inputs from different devices, thus promoting binaural fusion.

## Data Availability

Data will be made available on request.
